# Dig1 protects against locomotor and biochemical dysfunctions provoked by Roundup

**DOI:** 10.1186/s12906-016-1226-6

**Published:** 2016-07-22

**Authors:** Steeve Gress, Claire Laurant, Nicolas Defarge, Carine Travert, Gilles-Éric Séralini

**Affiliations:** University of Caen Normandy (UCN), Institute of Biology (IBFA), EA2608 and Network on Risks, Quality and Sustainable Environment MRSH, Esplanade de la Paix, CS 14032 Caen Cedex 5, France; Sevene Pharma, Mazet Pailhès, 30170 Monoblet, France

**Keywords:** Detoxification, Dig1 (Digeodren), Glyphosate-based herbicide, Kidney, Liver, Toxicity

## Abstract

**Background:**

Plant medicinal extracts may be claimed to prevent or cure chemical intoxications. Few of these are tested for their mechanisms of actions in vivo and for their cellular impacts. In 2011, we demonstrated that hepatic cell mortality induced by environmentally realistic levels of the widely used herbicide Roundup (R) in vitro can be almost entirely prevented by plant extracts called Dig1 (D, Digeodren).

**Methods:**

We tested the in vivo effects of D alone (1.2 ml/kg bw/d), but also prior to and during 8 days of R intoxication (at 135 mg/kg bw/d) in a total of 4 groups of 40 adult Sprague-Dawley male rats each. After treatments, horizontal and vertical locomotor activities of the animals were measured by use of actimeters. Brain, liver, kidneys, heart and testes were collected and weighted. Body weights as well as feed and water consumption were recorded. Proteins, creatinine, urea, phosphate, potassium, sodium, calcium, chloride ions, testosterone, estradiol, AST and ALT were measured in serum. In liver S9 fractions, GST, GGT, and CYP450 (1A2, 2C9, 2C19, 2D6, 3A4) were assessed.

**Results:**

D did not have any physiological or biochemical observable impact alone at 2 %. Out of a total of 29 measured parameters, 8 were significantly affected by R absorption within only 8 days. On these 8 parameters, only 2 were not restored by D (GGT activity and plasmatic phosphate), 5 were totally restored (horizontal and vertical locomotor activities, CYP2D6 activity, plasmatic Na + and estradiol), and the 6th was almost restored (plasmatic K+). The specificities of the toxic effects of R and of the therapeutic effects of D treatment were thus demonstrated, both at the behavioural and biochemical levels.

**Conclusions:**

D, without any side effect observable in these conditions, presented strong preventive and therapeutic properties in vivo after a short-term intoxication by the widely used pesticide Roundup.

**Electronic supplementary material:**

The online version of this article (doi:10.1186/s12906-016-1226-6) contains supplementary material, which is available to authorized users.

## Background

Plant medicinal extracts may be claimed to prevent or cure chemical intoxications. Few of these are tested for their mechanisms of action in vivo and for their cellular impacts. In 2011, we demonstrated [1] that hepatic cell mortality induced by environmental levels of the widely used herbicide Roundup (R) in vitro can be almost entirely prevented by plant extracts. The Dig1 (D) active mixture contained *Taraxacum officinalis*, *Arctium lappa* and *Berberis vulgaris*. We concluded that these properties should be confirmed in vivo. These herbal preparations were chosen in particular for their known digestive detoxification or hepato-protective effects [2–8]. *Taraxacum* is cited for protective effects in the digestive system [7, 8], as well as for anti-tumoral [9] and anti-oxidant effects [10]. *Arctium lappa* is also found to be hepato-protective [5, 6], as is *Berberis* [4]. In this study, we measured hepatic parameters, including various cytochrome P450 enzymes, blood parameters, and locomotor activities in the adult Sprague–Dawley rat after R and/or D treatments. The choice of this strain is in agreement with the U.S. National Toxicology Program [11]. We exposed young male 60 day-old adults to 0.5 % R, corresponding to half of the recommended agricultural dilution, comparable to an herbicidal spray.

Glyphosate-based pesticides, including all R formulations, are the most widely used non-selective herbicides. They are mixtures of glyphosate salts and co-formulants; the latter have been characterized as more toxic than glyphosate alone in various preparations and models [12, 13]. Glyphosate itself measured as a marker is one of the major surface water pollutants [14] and food contaminants in genetically modified plants, such as in Roundup tolerant soya [15], and is commonly found in human urine [16–18].

We know that these types of xenobiotics, which include corrosive adjuvants used as co-formulants, have a main endpoint in the liver, which is the major detoxification organ. We have previously demonstrated that very low levels of Roundup (0.1 ppb in tap water) exert endocrine-disrupting effects, such as sex hormone imbalances and hepatorenal toxicities, in mature rats after chronic exposure [19]. This was subsequently confirmed at a transcriptomic level [20]. We evaluated in this work whether it is possible to prevent R toxicity by D during short-term herbicide absorption, as if the animal were exposed to an agricultural spray. Prevention in vitro appeared to be quite effective at a cellular level, avoiding up to 1/3 or 1/4 of cellular toxic effects of R, when D is administered prior to intoxication. Thus in the present work we chose a protocol starting with D treatment before R exposure.

## Methods

### Animals, ethics and experimental design

**Fig. 1:**
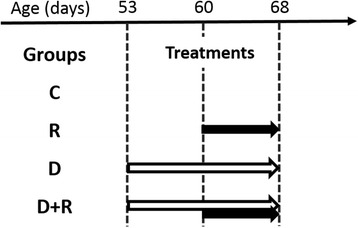
Experimental design. 4 groups of 40 mature male rats received the following treatments in drinking water: C control, R Roundup 0.5 %, D Dig1 2 %, D + R Dig1 2 % and Roundup 0.5 % (prevention and treatment). The lengths of the treatments are indicated by *arrows*

Care of animals complied with the recommendations of the Helsinki Declaration, and the study was performed in accordance with the regulations of the official edict of the French Ministry of Agriculture (A14-118-004) and with approval of the ethical committee (CENOMEXA N/01-01-13/01/01-16). In total, 160 male Sprague–Dawley rats (Janvier, Le Genest Saint-Isle, France), weighing 260–280 g, were fed and housed under standard conditions. The animals were maintained at 22 ± 3 °C under controlled humidity (45 to 65 %) and air purity with a 12 h-light/dark cycle, with free access to food (ref. 801151 RM1, Special Diet Services, UK) and water. The animals were randomized upon arrival, divided into 4 groups (4 × 40 animals, see Fig. 1 on experimental design) and kept in cages for 3 weeks. One was the control group, C; one was the first treated group, R, receiving in drinking water the glyphosate-based herbicide (GBH) Roundup GT Plus (R) diluted at a 0.5 % (recommended agricultural herbicide dilution 1–2 %) in a deionized water suspension (approximately 135 mg/kg body weight/day) for a short period (8 days from postnatal day 60). One other group, D, received Dig1 (D) added at 2 % in drinking water (1.2 ml/kg body weight/day) between the ages of 53 to 68 days, to test the effect of the detoxification mixture by itself on physiology. The last group, D + R, received in similar conditions D alone, administered preventatively (days 53–60), and then a mixture of D and R as a treatment during the next 8 days (60–68). The behaviour, blood and organs were then analysed. Animals were euthanized with sodium pentobarbital 125 mg/kg i.p. Biochemical tests were performed on 10 rats/group, while sexual hormones were measured in 20 rats/group. The body weight, water and food consumption were followed every 2 days for one week before the experiment and during the protocol period.

### Chemicals

D is a mixture of diluted organic plant extracts obtained by Sevene Pharma (Monoblet, France) from independent saturating macerates, corresponding to 1/10 of dried plants in a water-alcohol solution of 45 to 55 %. These are afterwards diluted in 70 % alcohol, with *Taraxacum officinalis* at 100 ppm (part per million), as well as for *Arctium lappa*, and *Berberis vulgaris* at 10 ppm. This mixture was previously studied in Gasnier et al. [1, 2]. The glyphosate-based herbicide (GBH) used was Roundup GT Plus (approval 202448, Monsanto), a commercial formulation composed of 607 g/L isopropylamine salt of glyphosate, equivalent to 450 g/L glyphosate acid, and formulants.

### Assessment of spontaneous locomotor activity by infrared photocell-based detection

In total, 24 animals per group were tested in the actimeters by the end of the treatment, according to methodologies detailed in Lynch et al. [21]. Briefly, before each session, the actimeters (Imetronic Neurosciences, Pessac, France) were checked for the correspondence between the number of manual infrared beam breaks and their recording. At the beginning of the activity test, the animals were individually placed into covered Plexiglas cages (38 × 24 × 21 cm international standards) inside a darkened enclosure containing 8 cages. Each cage was equipped with 4 infrared photocell units: 2 at each end of the cage and 3 cm above the bottom in order to assess movements within the horizontal plane, namely front and rear activities and back and forth shuttles, the sum of them being the horizontal activity. Then 10 additional photocell units placed 20 cm above the cage bottom at regular intervals allowed assessment of the vertical activity (rearing behaviour). The sum of these (total) was calculated. Each cage is connected to silent electronic counters (actimeters) and both horizontal and vertical activities were recorded by computer over a 30-min period at night.

### Organs and biochemical analyses

Brain, liver, kidneys, heart and testes were collected, weighted, and then stored at −80 °C. Samples of 10 mL of blood per animal were taken and stored at −20 °C. The blood samples were used to quantify proteins, creatinine, urea, phosphorus, potassium, sodium, calcium, chloride content and aspartate aminotransferase (AST), alanine aminotransferase (ALT) activities using the qualified Cobas Mira biochemistry analyser (4 M, France) according to the methods cited below. Creatinine was quantified using Jaffe procedure, urea by an enzymatic method based on Talke and Schubert reaction [22], calcium by photometry assay using metallo-chromogen Arsenazo III, and chloride by photometry assay using mercuric thiocyanate. Sodium level was determined via an enzymatic test by dosing of sodium dependent β-galactosidase activity with o-nitrophenyl-β-D-galactopyranoside as substrate. Potassium level was determined via an enzymatic test of potassium-dependent pyruvate kinase activity, using phosphoenolpyruvate as substrate. Inorganic phosphate was measured according to Daly and Ertinghausen [23] by a direct method quantifying unreduced phosphomolybdate heteropolyacid at 340 nm. ALT activity was determined by the method described by Wrobleski and LaDue [24] and optimized by Henri et al. [25] and Bergmeyer et al. [26], AST measurement was described by Karmen et al. [27], and optimized by Henri et al. [25].

S9 fractions from liver samples allowed assays of CYP450 activity, as well as of glutathione S-transferase (GST) and gamma-glutamyl transpeptidase (GGT). The CYP450 activities (1A2, 2C9; 2C19, 2D6 and 3A4) were evaluated by measuring the formation of specific CYP-dependent products (respectively acetaminophen, 4–hydroxydiclofenac, 4-hydroxymephenytoin, dextrorphan, 6β-hydroxytestosterone) following the addition of specific probes substrates (respectively phenacetin, diclofenac, S-mephenytoin, dextromethorphan and testosterone). These specific metabolites were analysed by reverse-phase liquid chromatography followed by electrospray ionization (ESI) in the positive mode and tandem mass spectrometry (MS/MS) detection. The GGT and GST activities were evaluated by kinetic assays. The GGT catalyses the transfer of the γ-glutamyl group from the substrate γ-glutamyl-3-carboxy-4-nitroanilide to glycylglycine, yielding 5-amino-2-nitrobenzoate. The change in absorbance at 405 nm is due to the formation of 5-amino-2-nitrobenzoate and is directly proportional to the GGT activity in the sample. Experiments were conducted in duplicate per rat liver S9 fraction, and the optical density was measured every 30 s during 20 min at 405 nm. GST catalyses the conjugation of reduced L-glutathione (GSH) to 1-chloro-2,4-dinitrobenzene substrate through the thiol group of the glutathione. The reaction product, GS-DNB conjugate, absorbs at 340 nm. The increase in the absorption is directly proportional to the GST activity in tested samples. Experiments were conducted in duplicate per rat liver S9 fraction, and the optical density was measured every 30 s during a period of 90 s at 340 nm.

Testosterone was measured using a colorimetric competitive immunoassay kit (EIA kit, Enzo, Villeurbanne, France). This assay is based on the competition between testosterone in the standard or the serum sample and a testosterone-alkaline phosphatase conjugate for limited amount of testosterone antiserum. The limit of detection is 5.67 pg/mL. The specificity of this assay is 100 % for testosterone and 14.6 % for 19 hydroxytestosterone. Samples were tested in duplicate and their concentrations were determined by comparing their respective absorbance values read at 405 nm, with those obtained for the reference standards plotted on a standard curve.

17β estradiol was measured using an enzyme-linked immunosorbent assay (17β estradiol high sensitivity ELISA kit, Enzo, Villeurbanne, France). This assay is based on the competition between 17β estradiol in the standard or the serum sample and a 17β estradiol-alkaline phosphatase conjugate for limited amount of 17β estradiol antiserum. The detection limit is 14.0 pg/mL. The specificity of this assay is 100 % for 17β estradiol and 17.8 % for estrone. Steroids in the serum samples were extracted with diethyl ether (5:1; v/v). Organic phases were dried down using a speed-vacuum dry for 2–3 h. Samples were then rehydrated at room temperature in an assay buffer and tested in duplicate. Their concentrations of 17β estradiol were determined by comparing their respective absorbance values, read at 405 nm, with those obtained for the reference standards plotted on a standard curve.

### Statistical analyses

All data were presented as the mean ± standard error of the mean (SEM) in scatter plots. Statistics were performed using GraphPad Prism 5 (GraphPad software, La Jolla, USA) software. Data were checked for Gaussian distribution by a Shapiro test and for homoscedasticity (Barlett’s test). In cases where these 2 conditions were met, the multiple comparisons test was an ANOVA followed by Bonferroni post-hoc test. In the other cases, statistical differences were determined by a non-parametric Kruskal-Wallis test followed by a Dunn’s post hoc test for multiple comparisons. Significant levels were reported with *p* < 0.05 (*), *p* < 0.01 (**) and *p* < 0.001 (***).

## Results

Among all parameters measured, only significant ones are reported below. In particular, no significant difference in food or water consumption was observed between the groups. Similarly, the body weights regularly and normally increased from arrival in the experimental environment to a mean of 570 ± 23 g after treatment.

### Locomotor activity

**Fig. 2:**
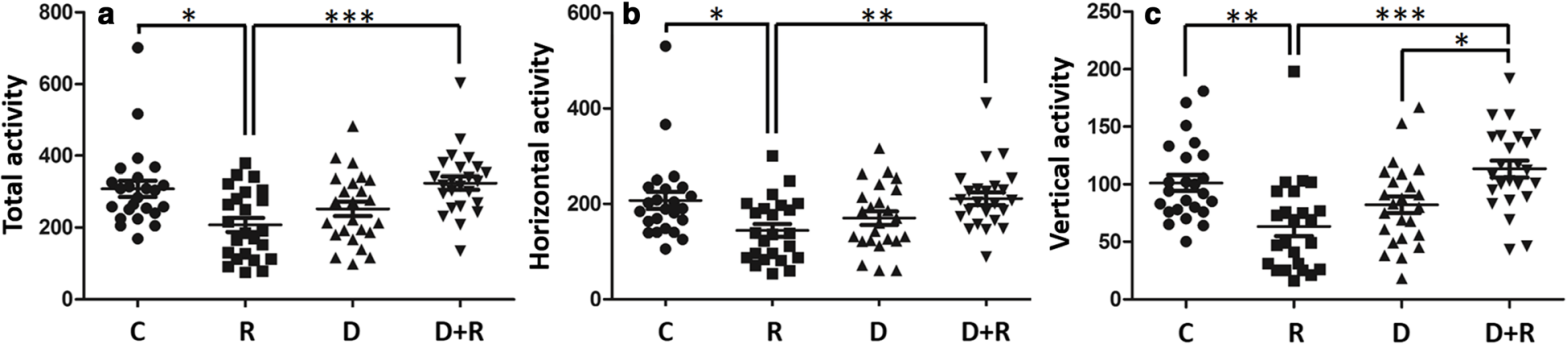
Locomotor activity of rats treated with Roundup and/or Dig1. Treatments are indicated in Fig. 1. Total activity (**a**) is the sum of horizontal activity (front, rear, back and forth shuttles, **b**) and vertical activity (rearing behaviour, **c**). Activities are measured over a period of 30 min by infrared photocell-based detection in an actimeter; counts correspond to infrared beam breaks. Significant differences in scatter plots (with mean ± SEM) were reported: *p* < 0.05 (*), *p* < 0.01 (**) and *p* < 0.001 (***)

As a general behaviour indication, the total locomotor activity (Fig. 2a) was significantly reduced in animals treated for only 8 days with R at 0.5 % (−33 %; *p* < 0.05). Both horizontal activity (−30 %; *p* < 0.05, Fig. 2b) and rearing or vertical behavior (−37 %; *p* < 0.01, Fig. 2c) were reduced similarly by the herbicide absorption (Fig. 2a, b, c). In contrast, no significant change in locomotor activity was noticed in animals receiving D, in comparison to controls. When animals were co-exposed to D and R, animals recovered normal horizontal and vertical locomotor activities. The therapeutic effect in comparison to R alone was very significant (+56 %, *p* < 0.001 for total activity, +46 %, *p* < 0.01 for horizontal activity, +112 %, *p* < 0.001 for rearing behavior).

### Organ weights and biochemical analyses

No significant changes were observed at this level except those described below. In any case, D alone had an effect in comparison to controls.

#### Liver parameters

**Fig. 3:**
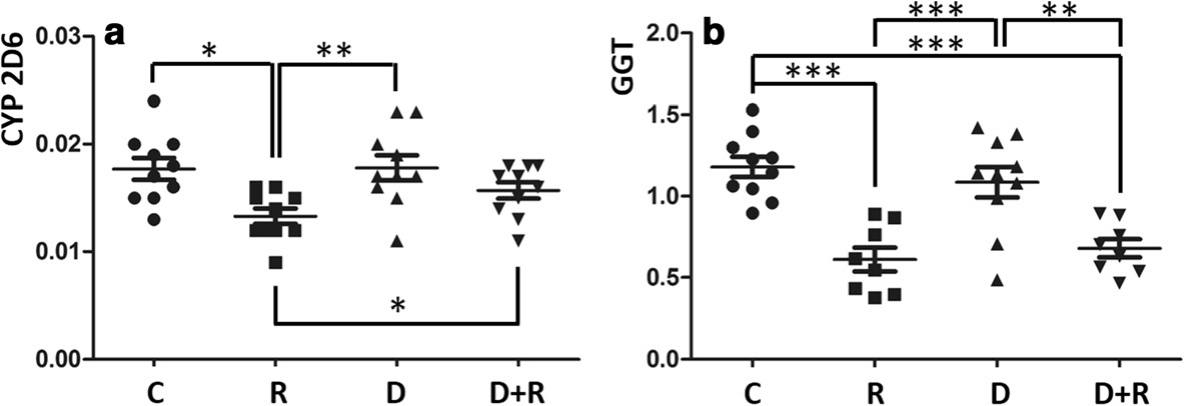
Activities of CYP2D6 and GGT of rats treated with Roundup and/or Dig1. Enzymatic activities were measured by the formation of specific products. CYP2D6 activity was monitored during a period of 1 h by tandem mass spectrometry. Results are expressed by the ratio area of the analyte/area of the internal standard (**a**). GGT activity was measured by spectrophotometry at 405 nm in unit/mg protein (**b**). Significant differences in scatter plots (with mean ± SEM) were reported: *p* < 0.05 (*), *p* < 0.01 (**) and *p* < 0.001 (***)

R treatment decreased CYP2D6 activities (−25 %, *p* < 0.05, Fig. 3a) and GGT (−48 %, *p* < 0.001, Fig. 3b). These inhibitions were restored only for CYP2D6 with D added in prevention and co-treatment (+18 %, p < 0.05, one-tailed *t*-test). GGT was not affected by D.

#### Kidney parameters

**Fig. 4:**
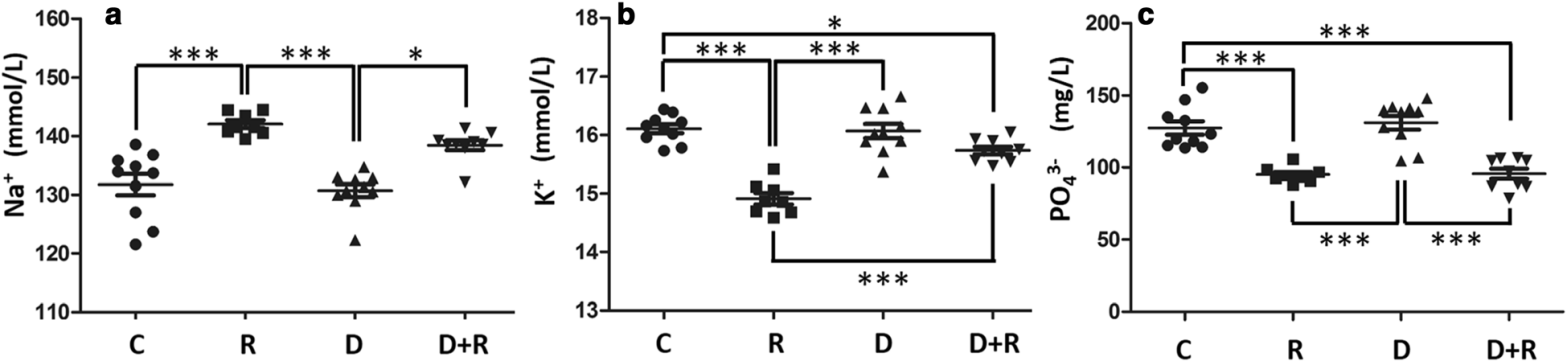
Plasmatic levels of ions of rats treated with Roundup and/or Dig1. Sodium levels in mmol/L (**a**) were determined via an enzymatic test of sodium dependent β-galactosidase activity with o-nitrophenyl-β-D-galactopyranoside as substrate. Potassium levels in mmol/L (**b**) were determined by dosing potassium-dependent pyruvate kinase activity using phosphoenolpyruvate as substrate. Inorganic phosphate in mg/L (**c**) was measured by a direct method quantifying unreduced phosphomolybdate heteropolyacid at 340 nm. Significant differences in scatter plots (with mean ± SEM) were reported: *p* < 0.05 (*), *p* < 0.01 (**) and *p* < 0.001 (***)

R treatment increased serum Na + (+7.8 %, *p* < 0.001, Fig. 4a), and decreased K+ (−7.4 %, *p* < 0.001, Fig. 4b) and phosphate (−25 %, *p* < 0.001, Fig. 4c). D had a tendency to re-equilibrate plasmatic Na + and K+, though not totally, but D did not affect the phosphate imbalance provoked by R.

#### Sex hormone levels

**Fig. 5:**
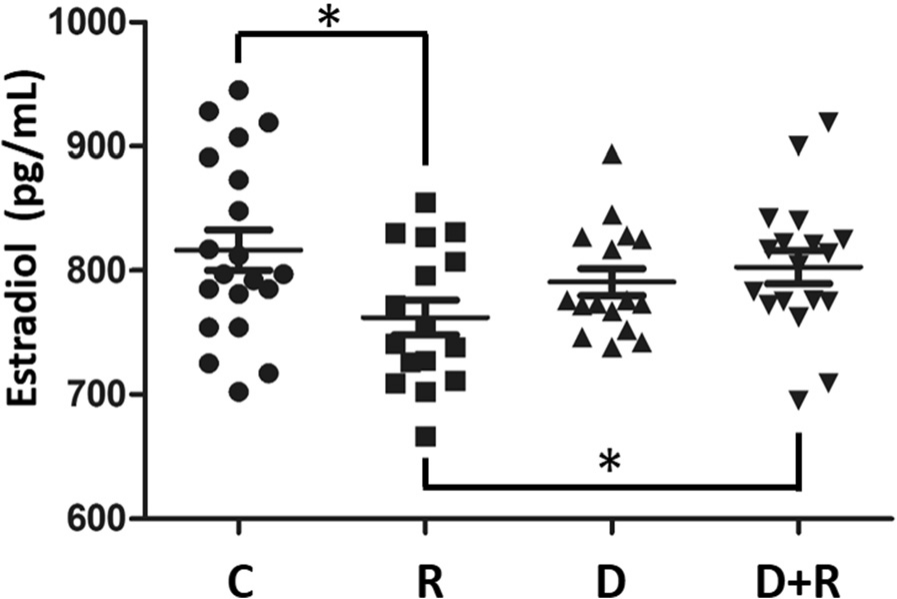
Plasmatic levels of estradiol of rats treated with Roundup and/or Dig1. 17-β estradiol levels in pg/mL were measured using an enzyme-linked immunosorbent assay. Significant differences in *scatter plots* (with mean ± SEM) were reported: *p* < 0.05 (*), *p* < 0.01 (**) and *p* < 0.001 (***)

R treatment decreased estradiol (−6.7 %, *p* < 0.05, Fig. 5) but not testosterone levels. The low plasmatic estradiol decrease was compensated by D (+5.3 %, *p* < 0.05, one-tailed *t*-test) up to control levels.

## Discussion

On a total of 29 parameters measured in this study and described in materials and methods, 8 were significantly affected by R absorption in 8 days only. Out of these 8 parameters, only 2 were not restored by D (liver GGT activity and plasmatic phosphate). Indeed, 5 were totally restored (horizontal and vertical locomotor activities, liver CYP2D6 activity, plasmatic Na+, estradiol, and the 6th almost (plasmatic K+). This demonstrates the specificity of the toxic effect of R and of the treatment on precise parameters, both at a biochemical and behavioural level. Moreover, D by itself did not have any observable physiological or biochemical impact at 2 %, on all parameters measured, confirming findings in vitro [1, 2].

Locomotor activity assessment by actimeters is useful and validated for a global evaluation of the toxicological effect [21]. Rapid changes in behaviour reflect the impact of chemicals on various physiological functions and the general health status. As an example, pollutants and specifically pesticides may induce neurological and behavioural disturbances [28–31], including depression [32], or longer-term degenerative diseases like Parkinson’s or Alzheimer’s pathologies [33, 34]. In this experiment, R reduced significantly and importantly (−33 %) both the horizontal and rearing movements. When these animals were treated with D before (for prevention) and during the 8-day intoxication with R, locomotor activities were restored to control values. This trend was consistent across the other affected parameters. R could have impacted neurotransmission, since it has been shown to be a dopamine inhibitor in male rats, leading to hypoactivity [35]. R is an aromatase inhibitor [36] and an endocrine disruptor [19], aromatase being the enzyme that synthesizes estrogens. Endocrine disruptors like synthetic estrogens may be also nervous system disruptors [37]; thus they may impinge on the metabolism and binding of dopamine, the neurotransmitter stimulating exploratory behaviour [38]. In other models, aromatase inhibitors are known to modulate the dopaminergic system [39]. The restoration by D of R-induced dopamine inhibition could be explained by the fact that berberine (in *Berberis vulgaris*) prevents nigrostriatal dopaminergic neuronal loss in mice [40].

Another target of toxicity may be the heart and other muscles, which present contraction difficulties after R treatment; this could explain the locomotor mitigations. R impacted cardiac excitability in the rat ventricular myocardium [41], which is modulated by the Na+/K+ balance. The latter has been modified in the plasma in this work (see below). In fact, glyphosate-based herbicides such as R potently affect the cardiovascular system in mammals [42]. Arctigenin present in D (in *Arctium lappa*) activates muscle uptake of glucose [43], which could restore the locomotor activity. Berbamine (in *Berberis vulgaris*) may modulate myocardial contractility [44]. *Taraxacum officinalis* has some anti-fatigue properties [45]. The kidney itself in the adult [19], as well as renal embryonic cells [1, 46], are sensitive to R toxicity. Renal dysfunction is also able to affect the general behavior. The protection of kidney cells by D has been previously documented [1, 2].

CYP2D6 is the major cytochrome P450, among the 5 measured in this work, which is modulated by an R specific inhibition. It is expressed in the liver and central nervous system, and is one of the most important enzymes in xenobiotic metabolism and neurosteroids [47]. It may be inhibited by pesticides [48] and only a few substances are known to increase or restore its levels, as D was demonstrated to do in this work. This effect has not been observed for D before. Interestingly, GGT, which is used in the detoxification of some xenobiotics, is also inhibited by R but not restored by D, showing the specificity of the treatment effect.

For plasmatic ions, R induces a Na+/K+ imbalance with an equivalent increase in Na + and decrease in K+ (around 7–8 % for both). This reflects a possible kidney injury, already noticed for R [19] and confirmed by a phosphate loss. The compensation effect is again detected for D at the Na+/K+ level, but not for phosphates. In general, polyphenolic compounds present in D are considered liver or kidney protective [49–51]. For instance, berberine in D protects renal proximal tubular cells from mitochondrial stress and apoptosis [52], which are known to be induced by R [46].

A plasmatic decrease in estradiol by R in this work confirms the aromatase inhibition provoked by R. There is less testosterone synthesis observed in testicular rat cells after R treatment [53]. The plasmatic decrease of this aromatase substrate could also disturb estradiol production and have an impact on the androgen/estrogen balance essential for reproduction. In vivo the testicular aromatase inhibition was demonstrated after 8 days of R toxicity [54]. Berberine (in *Berberis vulga*ris) may modulate estradiol production through 17b-HSD enhancement [55]; this could explain the compensatory effect observed after D treatment.

## Conclusion

This is a first demonstration of a Dig1 protective effect in vivo after intoxication by a major pesticide. This study can be supplemented by testing other concentrations of Dig1 and multiple brain, nervous, cardiac, hepatic and renal parameters, but also after longer intoxications by Roundup (R) and for other pollutants. Our results evidence the reversal, by specific plant extracts, of some of the adverse effects provoked by R, especially on general health, as measured by locomotor activity. Most biochemical disturbances due to R were also reversed by Dig 1 administered prior and together with R.

## Abbreviations

ALT, alanine aminotransferase; AST, aspartate aminotransferase; bw, body weight; CYP, cytochrome P450; d, day; D, Dig1, Digeodren; ELISA, enzyme linked immunosorbent assay; ESI, electrospray ionization; GBH, glyphosate-based herbicides; GGT, gamma-glutamyl transpeptidase; GS-DNB, glutathione-dinitrobenzene; GSH, L-glutathione; GST, glutathione S-transferase; MS/MS, tandem mass spectrometry; ppb, part per billion; R, Roundup; SEM, standard error of the mean

## Additional file

Additional file 1: Raw data for locomotor activity and biochemical parameters of rats treated with Roundup (R), Dig1 (D), Dig1+Roundup (D+R) in comaprison to controls (C). Excel sheet1: front activity (in counts); 2: rear activity (in counts); 3: sum "front+rear" (in counts); 4: shuttles back and forth (in counts); 5: horizontal activity (sum "front+rear+shuttles back and forth", in counts); 6: vertical activity or rearing behavior (in counts); 7: total locomotor activity (sum "horizontal+vertical" activities); 8: CYP2D6 activity in liver S9 fraction (difference between T1h and T0 of the ratio "area of the analyte/area of the internal standard" x 100); 9: GGT activity (U/mg protein) in liver S9 fraction; 10: sodium plasmatic level (mmol/L); 11: potassium plasmatic level (mmol/L); 12: inorganic phosphate plasmatic level (mg/L); 13: estradiol plasmatic level (pg/mL). (XLSX 29 kb)
